# Supplementary material for: Diverging trends and drivers of Arctic flower production in Greenland over space and time
Source: Polar Biol. 2023 Jul 12;46(9):837–48. doi: 10.1007/s00300-023-03164-2 (PMC10425507; doi:10.1007/s00300-023-03164-2)
Supplement: Supplementary file 1 — Supplementary file1 (DOCX 2930 KB) [file 300_2023_3164_MOESM1_ESM.docx]

**SUPPLEMENTARY INFORMATION**

Diverging trends and drivers of Arctic flower production in Greenland over space and time

**Antoine Becker-Scarpitta***^1,2,3^, Laura H. Antão^4^, Niels Martin Schmidt^5,6^, F. Guillaume Blanchet^7,8,9^ Elina Kaarlejärvi^4^, Katrine Raundrup^10^, Tomas Roslin^1,4,11^

1: Department of Agricultural Sciences, University of Helsinki, Helsinki, Finland.

2: Institute of Botany of the Czech Academy of Sciences, Brno, Czech Republic.

3: CIRAD, UMR PVBMT, F-97410 Saint Pierre, La Réunion, France.

4: Research Centre for Ecological Change, University of Helsinki, Helsinki, Finland.

5: Department of Ecoscience, Aarhus University, Roskilde, Denmark.

6: Arctic Research Centre, Aarhus University, Aarhus, Denmark.

7: Département de biologie, Université de Sherbrooke, Sherbrooke, QC, Canada.

8: Département de mathématiques, Université de Sherbrooke, Sherbrooke, QC, Canada.

9: Département des sciences de la santé communautaire, Université de Sherbrooke, Sherbrooke, QC, Canada.

10: Department of Environment and Mineral Resources, Greenland Institute of Natural Resources, Nuuk, Greenland.

11: Department of Ecology, Swedish University of Agricultural Sciences, Uppsala, Sweden.

*corresponding author: antoine.becker.scarpitta@gmail.com

# Table of content

[**Online resource 1 - Details of the study area and the location of the plots**](#_heading=h.pcmz96o5ox3w) **3**

[**Online resource 2 – Sampling design used to generate time series of flower abundances.**](#_heading=h.ifca8tg5ajqg) **7**

[**Online resource 3 – Ecological details of the species studied.**](#_heading=h.z6pg1mu8xo67) **8**

[**Online resource 4 - Climatic trends of Precipitation (mm), Temperature (°C) and Snowmelt day**](#_heading=h.gjdgxs) **9**

# Online resource 1 - Details of the study area and the location of the plots

**Figure S1 - Map of the study sites:** (a) the low-Arctic site (Southwest Greenland, 64° 08’ N, 51° 23’ W - Kobbefjord research station; Raundrup, Olsen, et al. 2020) and (b) the high-Arctic site (Northeast Greenland, 74° 29’ N, 21° 34’ W - Zackenberg research station; Schmidt, Hansen, et al. 2019a). Dots indicate the permanent monitoring plots where flower densities were recorded, with abbreviations identifying the focal plant species: CAS=*Cassiope tetragona*, DRY=*Dryas integrifolia×octopetala*, ERI=*Eriophorum angustifolium*, LOI=*Loiseleuria procumbens*, PAP=*Papaver radicatum*, SAX=*Saxifraga oppositifolia*, SAL=*Salix* spp. (with the species monitored being *Salix glauca* at Nuuk and *Salix arctica* at Zackenberg), SIL=*Silene acaulis*, and number indicating the plot ID. Further details are shown in Table S1.

**
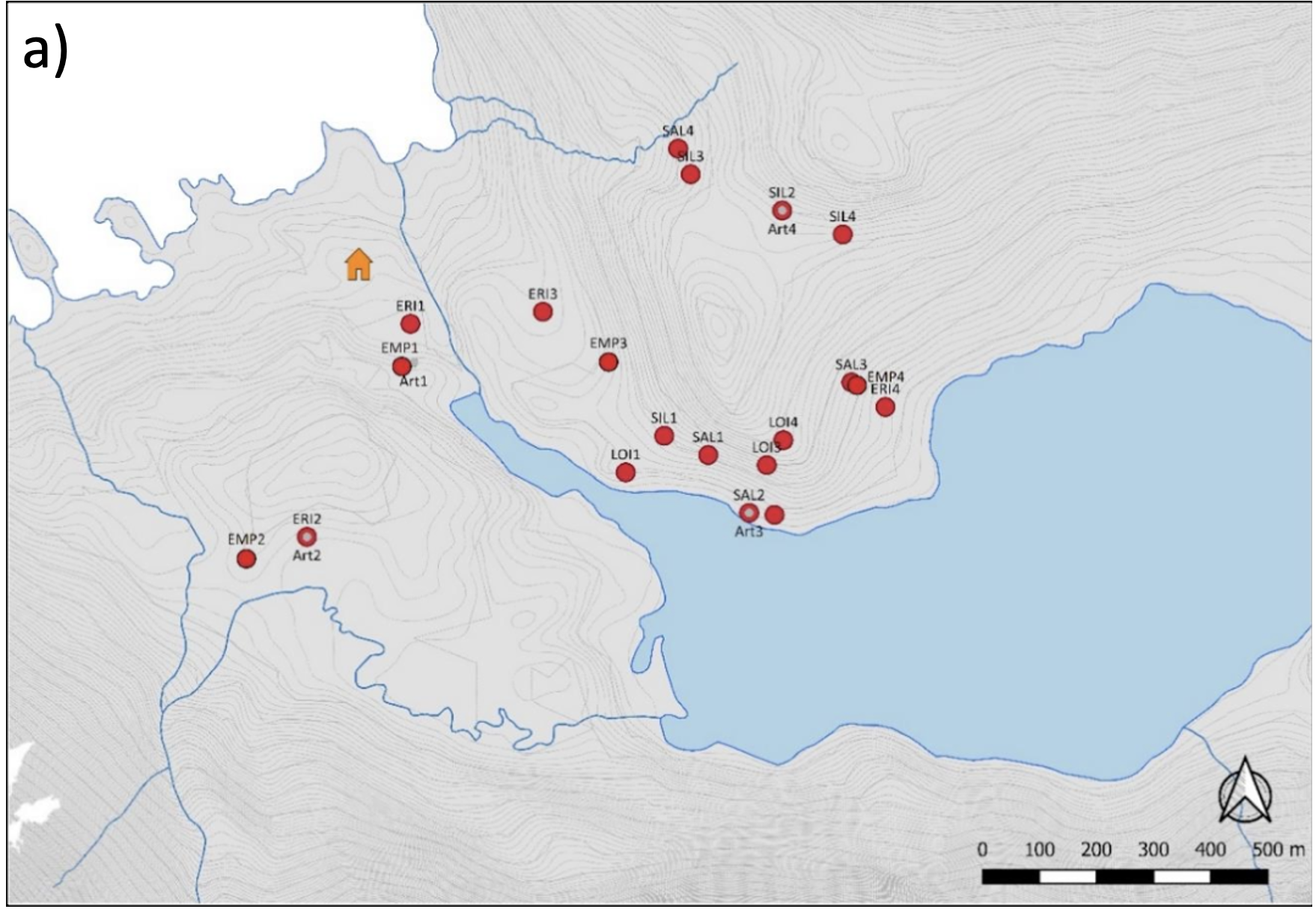
**

**
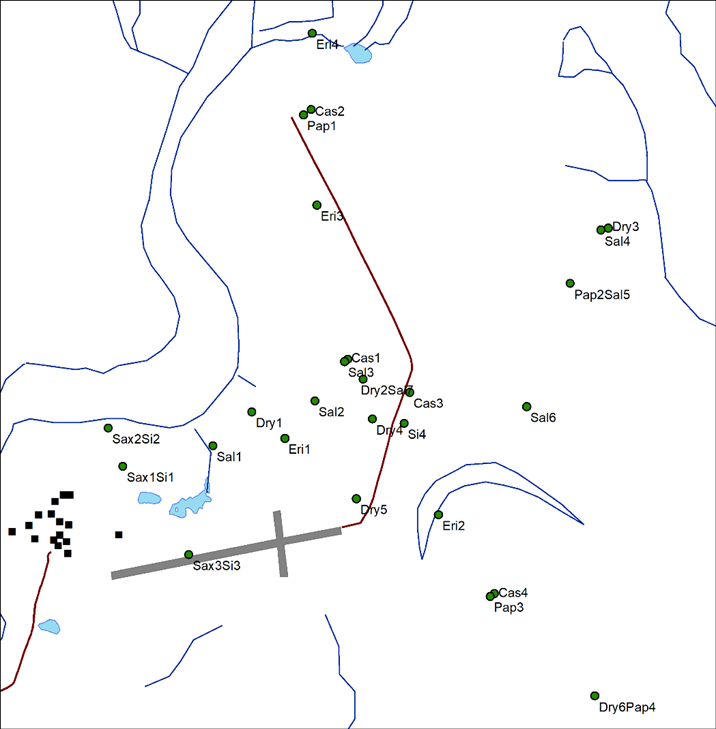
**

**Table S1 - Details of each plot at the low-Arctic and the high-Arctic site: shown are** GPS coordinates and the dimension of each plot.

| **Plots in the high-Arctic site** | | | | |
| --- | --- | --- | --- | --- |
| **Species** | **Plot** | **UTM E** | **UTM N** | **Plot dimensions (m)** |
| *Cassiope tetragona* | CAS1 | 513,3 | 8,264,809 | 1x2 |
|  | CAS2 | 513,23 | 8,265,290 | 1x3 |
|  | CAS3 | 513,42 | 8,264,746 | 1x2 |
|  | CAS4 | 513,583 | 8,264,358 | 1x3 |
| *Dryas integrifolia×octopetala* | DRY1 | 513,116 | 8,264,708 | 1x4 |
|  | DRY2 | 513,33 | 8,264,771 | 6x 10 |
|  | DRY3 | 513,802 | 8,265,062 | 1x2 |
|  | DRY4 | 513,348 | 8,264,694 | 2x3 |
|  | DRY5 | 513,317 | 8,264,541 | 2x3 |
|  | DRY6 | 513,776 | 8,264,161 | 7x 13 |
| *Papaver radicatum* | PAP1 | 513,215 | 8,265,280 | 7x 15 |
|  | PAP2 | 513,729 | 8,264,955 | 10x 15 |
|  | PAP3 | 513,575 | 8,264,353 | 9x 10 |
|  | PAP4 | 513,776 | 8,264,161 | 7x 13 |
| *Saxifraga oppositifolia* | SAL1 | 513,041 | 8,264,643 | 6x 10 |
|  | SAL2 | 513,237 | 8,264,729 | 15x 20 |
|  | SAL3 | 513,294 | 8,264,805 | 6x6 |
|  | SAL4 | 513,788 | 8,265,058 | 10x 15 |
|  | SAL5 | 513,729 | 8,264,955 | 10x 15 |
|  | SAL6 | 513,645 | 8,264,718 | 10x 15 |
|  | SAL7 | 513,33 | 8,264,771 | 6x 10 |
| *Salix arctica* | SAX1 | 512,867 | 8,264,603 | 2x3.5 |
|  | SAX2 | 512,839 | 8,264,677 | 2x3 |
|  | SAX3 | 512,994 | 8,264,433 | 2x5 |
| *Silene acaulis* | SIL1 | 512,867 | 8,264,603 | 2x3.5 |
|  | SIL2 | 512,839 | 8,264,677 | 2x3 |
|  | SIL3 | 512,994 | 8,264,433 | 2x5 |
|  | SIL4 | 513,409 | 8,264,686 | 1x1 |
| **Plots in the low-Arctic site** | | | | |
| **Species** | **Plot** | **Latitude** | **Longitude** | **Plot dimensions (m)** |
| *Eriophorum angustifolium* | ERI1 | 64.1346 | -51.3837 | 4 x 10 |
|  | ERI2 | 64.1312 | -51.3873 | 10 x 10 |
|  | ERI3 | 64.1348 | -51.3789 | 12 x 17 |
|  | ERI4 | 64.1333 | -51.3666 | 5 x 9 |
| *Salix glauca* | SAL1 | 64.1325 | -51.3729 | 7 x 11 |
|  | SAL2 | 64.1316 | -51.3714 | 8 x 8 |
|  | SAL3 | 64.1337 | -51.3678 | 6 x 9 |
|  | SAL4 | 64.1374 | -51.3741 | 4 x 5 |
| *Silene acaulis* | SIL4 | 64.1361 | -51.3681 | 5 x 12 |
|  | SIL1 | 64.1328 | -51.3745 | 5.5 x 7 |
|  | SIL2 | 64.1364 | -51.3703 | 11 x 11 |
|  | SIL3 | 6.4137 | -51.3736 | 7 x 11 |
| *Loiseleuria procumbens* | LOI1 | 64.1323 | -51.3759 | 1.8 x 3.9 |
|  | LOI2 | 64.1316 | -51.3705 | 1.7 x 3 |
|  | LOI3 | 64.1324 | -51.3708 | 1.6 x 2.6 |
|  | LOI4 | 64.1328 | -51.3702 | 1.6 x 3 |

# Online resource 2 – Sampling design used to generate time series of flower abundances.

Shown are the number of plots in which the respective plant species was monitored in a given year. Species names are abbreviated as in Appendix 1. Overall, sampling at the high-Arctic site of Zackenberg has continued for 25 years, and sampling at the low-Arctic site Nuuk has continued for 13 years.


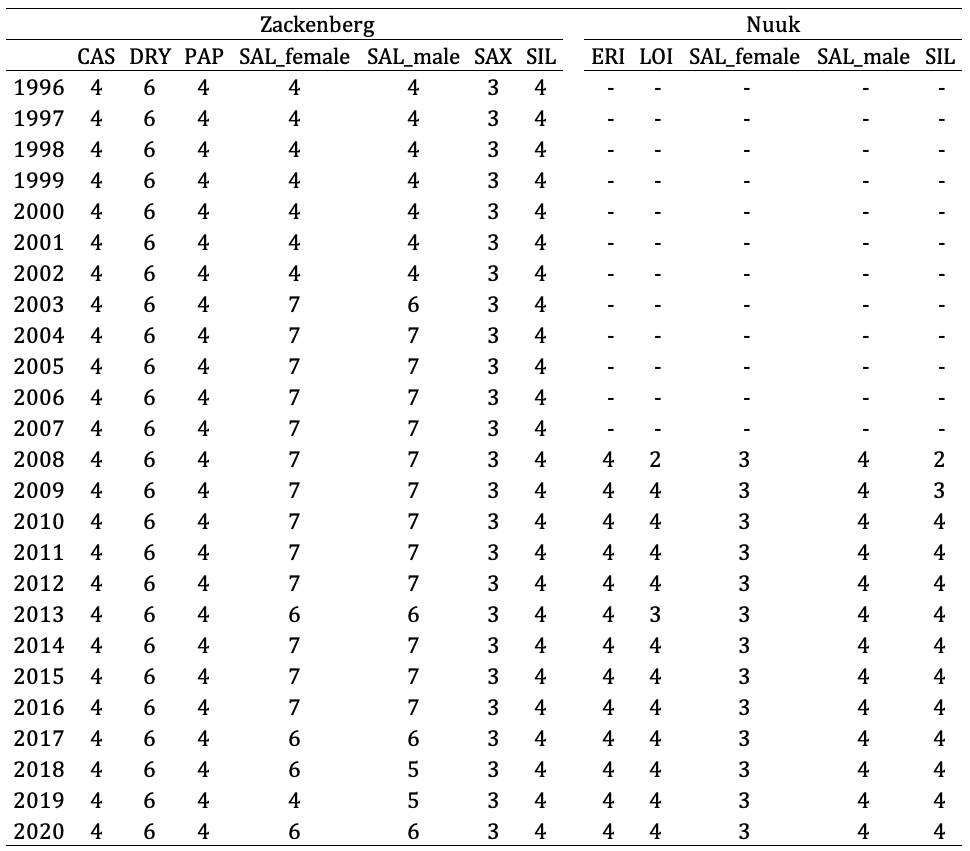


# **Online resource 3 – Ecological details of the species studied**.

Due to the advanced development of buds at the end of the previous growing season, *Saxifraga oppositifolia* L. is the earliest species to flower during spring in May-June (Stenström, et al 1997). It is a densely herbaceous succulent plant, both solitary and mat-forming. It produces cushions by shorter branches or forms extensive mats by long prostrate perennial branches, common on different soil types.

Species flowering in June-July are: *Loiseleuria procumbens* (L.) Desvaux, *Salix arctica* L., *Salix glauca* L., *Eriophorum angustifolium* L., and *Papaver radicatum* Rottb. Of these, *Loiseleuria procumbens* is an evergreen prostrate dwarf-shrub, growing on acid and dry heaths. *Salix arctica* is a short-statured shrub with a large ecological niche widely distributed across the Arctic heath and fell fields. *Salix glauca* is a deciduous shrub that typically grows on sand and cobbles among granitic boulders, sandy alluvium or scree slopes. *Eriophorum angustifolium* is a perennial graminoid, commonly found on peaty wet soil; and *Papaver radicatum* is a hardy forb forming tufts. It is a generalist species widely distributed across the Arctic fell fields.

*Cassiope tetragona* (L.) D. Don, *Silene acaulis* (L.) Jacq are the latest species to flower in July-August. Of these, *Cassiope tetragona* is an evergreen dwarf shrub with a large ecological niche, whereas *Silene acaulis* is a forb which grows in compact cushions and occurs on sandy open fell fields and heathland.

Finally, the hybrid *Dryas integrifolia×octopetala* is a cushion-forming evergreen shrub, common in non-acidic Arctic tundra heath. It has a very broad flowering niche from June to August.

# **Online resource 4 - Climatic trends of Precipitation (mm), Temperature (°C) and Snowmelt day**

at the low-Arctic site for the period 2007-2020 and the high-Arctic sites for the period 1995-2020.

**Figure S4a: Mean annual trends for Precipitation and Temperature.** Mean annual precipitation increased in the high Arctic (*β*=0.001±SE0.0003, *t*=3.42, *p*=0.002), while it decreased in the low Arctic (*β*= -0.003±0.002, *t*=-1.93, *p*=0.04). We detected significant warming in the high Arctic (*β*=0.067±0.02, *t*=3.52, *p*=0.001), while no change in annual temperature was detected in the low Arctic (*β=*0.16±0.14*, t*=1.12, *p*=0.3).


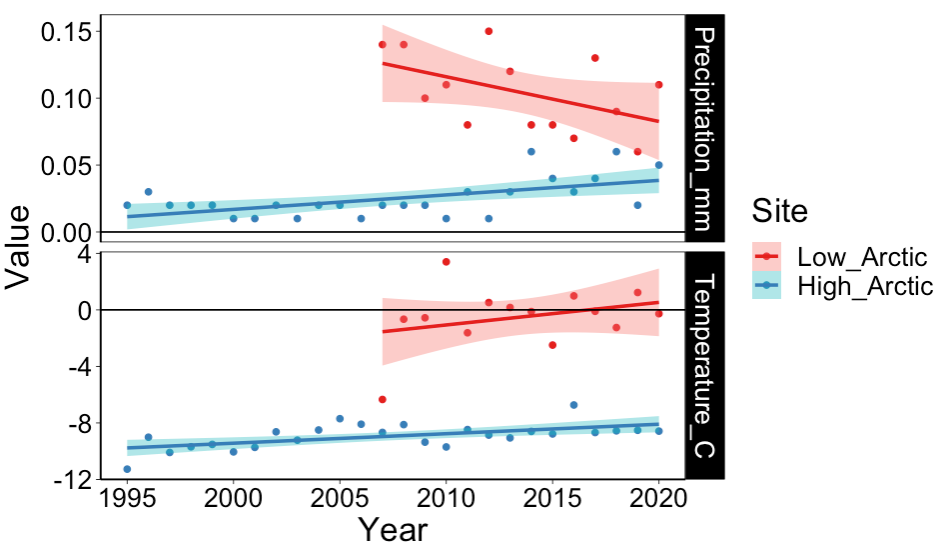


**Figure S4b: Mean seasonal trends of Precipitation and Temperature,** with *Summer=*June to August, and *Autumn*=September to November. Exact trend estimates are given in Table S4b.


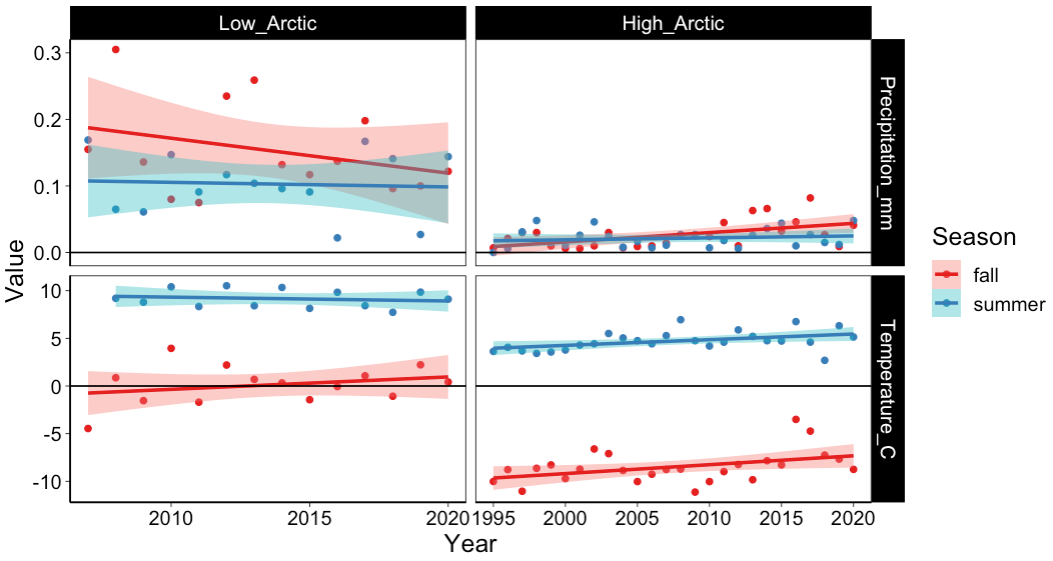


**Table S4a: Temporal trends in mean seasonal temperature and precipitation** in the high- (a) and low- (b) Arctic sites. Bold values are significant.

|  | *Estimate* | *std.error* | *t* | *p* |
| --- | --- | --- | --- | --- |
| **a) High Arctic** |  |  |  |  |
| **Precipitation** |  |  |  |  |
| (Intercept) | -1.67 | 0.615 | -2.72 | **0.009** |
| Year:Season_fall | 0.001 | 0.0003 | 2.76 | **0.008** |
| Year:Season_summer | 0.001 | 0.0003 | 2.75 | **0.008** |
| **Temperature** |  |  |  |  |
| (Intercept) | -155.5 | 47.9 | -3.25 | **0.002** |
| Year:Season_fall | 0.073 | 0.024 | 3.07 | **0.003** |
| Year:Season_summer | 0.08 | 0.024 | 3.35 | **0.001** |
| **b) Low Arctic** |  |  |  |  |
| **Precipitation** |  |  |  |  |
| (Intercept) | 6.11 | 5.65 | 1.08 | 0.3 |
| Year:Season_fall | -0.003 | 0.003 | -1.06 | 0.3 |
| Year:Season_summer | -0.003 | 0.003 | -1.06 | 0.3 |
| **Temperature** |  |  |  |  |
| (Intercept) | -105.43 | 193 | -0.635 | 0.5 |
| Year:Season_fall | 0.052 | 0.082 | 0.640 | 0.5 |
| Year:Season_summer | 0.057 | 0.082 | 0.695 | 0.5 |

**Figure S4c**: **Temporal trends of Snowmelt day of the year** in the low- (left) and high-Arctic (right) sites. The snowmelt day of the year was calculated as the date when 50% of the plot reached 50% of snow cover. No significant trends were found in the low Arctic, while all plots (each species was inventoried in an independent set of plots, Online resource S1) showed a significant decrease in the snowmelt day in the high Arctic, meaning earlier melting of snow. Exact trend estimates are given in Table S4c.

DOY-120=30 April; DOY-140=20 May; DOY-160=9 June; DOY-180=29 June.


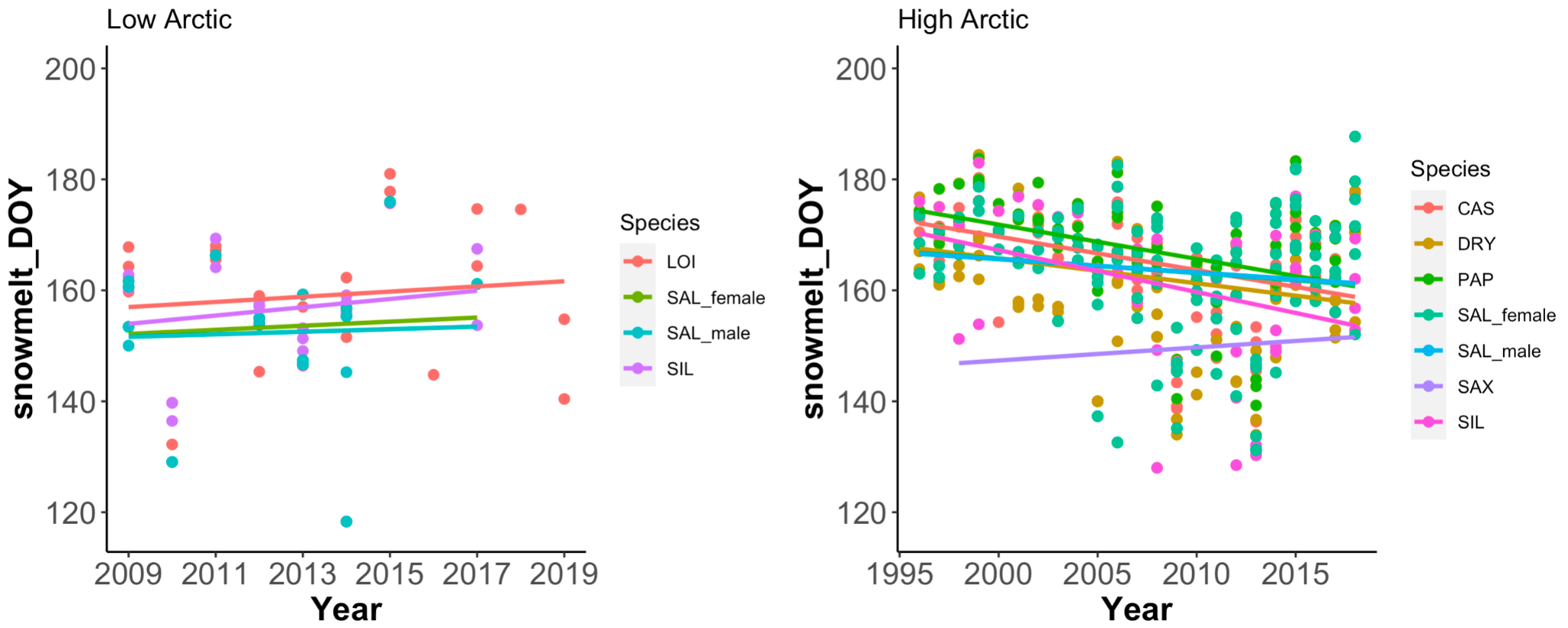


**Table S4b: Temporal trends of Snowmelt day of the year for each species plots** in the high- (a) and low- (b) Arctic sites. Bold values are significant.

|  | *Estimate* | *std.error* | *t* | *p* |
| --- | --- | --- | --- | --- |
| **a) High Arctic** |  |  |  |  |
| Year:Species_CAS | -0.603 | 0.141 | -4.28 | **<0.001** |
| Year:Species_DRY | -0.447 | 0.182 | -2.45 | **0.01** |
| Year:Species_PAP | -0.619 | 0.147 | -4.21 | **<0.001** |
| Year:Species_SAL_female | -0.260 | 0.177 | -1.47 | 0.1 |
| Year:Species_SAL_male | -0.240 | 0.179 | -1.34 | 0.1 |
| Year:Species_SAX | 0.235 | 0.476 | 0.493 | 0.6 |
| Year:Species_SIL | -0.755 | 0.309 | -2.44 | **0.01** |
| **b) Low Arctic** |  |  |  |  |
| Year:Species_LOI | 0.466 | 0.875 | 0.544 | 0.592 |
| Year:Species_SAL_female | 0.369 | 1.65 | 0.224 | 0.826 |
| Year:Species_SAL_male | 0.230 | 1.39 | 0.165 | 0.871 |
| Year:Species_SIL | 0.747 | 0.859 | 0.869 | 0.395 |
